# Acute exacerbation of Hashimoto's thyroiditis in a patient treated with dimethyl fumarate for multiple sclerosis: A case report: Erratum

**DOI:** 10.1097/MD.0000000000016555

**Published:** 2019-07-19

In the article, “Acute exacerbation of Hashimoto's thyroiditis in a patient treated with dimethyl fumarate for multiple sclerosis: A case report”,^[1]^ which appeared in Volume 98, Issue 17 of *Medicine*, 1b needs to be 1a in two sentences in the Case section. Theese sentence should appear as “She was previously treated for 3 years with interferon-β (IFN-β) 1a 22 mg subcutaneously 3 times a week from which she was switched to DMF due to flu-like syndrome side effects” and “During treatment with IFN-β 1a as well as before beginning treatment with DMF, findings of thyroid function test were within normal limits, with positive antithyroid antibodies (Table 1).”
